# Supplementary material for: Air temperature and diet influence body composition and water turnover in zoo-living African elephants (Loxodonta africana)
Source: R Soc Open Sci. 2020 Nov 25;7(11):201155. doi: 10.1098/rsos.201155 (PMC7735349; doi:10.1098/rsos.201155)
Supplement: Table S1 [file rsos201155supp1.docx]

**Table S1.** Overview of measurements for each subject at each time point. Data are presented on average temperatures, sex, age, body mass, TBW, FFM, body fat, water turnover, dilution space (N) and deuterium depletion rate (kD).

| Month-Year | Mean Temp. (C°) | Min  Temp.  (C°) | Max.  Temp.  (C°) | Dew point (C°) | Subject | Sex | Age | Mass (kg) | TBW (L)^a^ | FFM (kg)^a^ | Body Fat% | Water Turnover (L/d) | N | kD |
| --- | --- | --- | --- | --- | --- | --- | --- | --- | --- | --- | --- | --- | --- | --- |
| Jan-15 | 6 | -3 | 7 | -2 | 1770 | M | 32 | 5785 | 3772 | 5056 | 13 | 301 | 375.26 | 0.0758 |
| Mar-15 | 12 | 7 | 17 | 4 | 27 | M | 41 | 5626 | 3751 | 5029 | 11 | 319 | 379.90 | 0.0809 |
| Aug-15 | 24 | 19.4 | 28.8 | 18.3 | 1770 | M | 32 | 5591 | 3553 | 4763 | 15 | 421 | 380.84 | 0.1127 |
| Aug-15 | 24 | 19.4 | 28.8 | 18.3 | 1772 | F | 13 | 2556 | 1690 | 2265 | 11 | 271 | 358.66 | 0.1523 |
| Aug-15 | 24 | 19.4 | 28.8 | 18.3 | 27 | M | 41 | 5595 | 3439 | 4610 | 18 | 553 | 382.35 | 0.1530 |
| Aug-15 | 24 | 19.4 | 28.8 | 18.3 | 1611 | F | 33 | 4135 | NA | NA | NA | NA | NA | NA |
| Aug-15 | 24 | 19.4 | 28.8 | 18.3 | 1771 | F | 37 | 3756 | 2410 | 3231 | 14 | 267 | 381.39 | 0.1054 |
| Mar-16 | 11 | 4.4 | 17.2 | 3.9 | 1770 | M | 33 | 5744 | 4116 | 5518 | 4 | 329 | 338.96 | 0.0759 |
| Mar-16 | 11 | 4.4 | 17.2 | 3.9 | 1772 | F | 14 | 2578 | 1640 | 2199 | 15 | 233 | 398.13 | 0.1348 |
| Mar-16 | 11 | 4.4 | 17.2 | 3.9 | 1611 | F | 33 | 4130 | NA | NA | NA | NA | NA | NA |
| Mar-16 | 11 | 4.4 | 17.2 | 3.9 | 27 | M | 42 | 5425 | 3717 | 4982 | 8 | 318 | 347.86 | 0.0812 |
| Mar-16 | 11 | 4.4 | 17.2 | 3.9 | 1771 | F | 38 | 3805 | 2536 | 3399 | 11 | 313 | 378.78 | 0.0872 |
| Sep-16 | 23 | 19.4 | 26.7 | 20.0 | 1770 | M | 33 | 5903 | 4443 | 5956 | -1 | 356 | 337.73 | 0.0763 |
| Sep-16 | 23 | 19.4 | 26.7 | 20.0 | 1772 | F | 14 | 2654 | 1789 | 2398 | 10 | 256 | 381.07 | 0.1359 |
| Sep-16 | 23 | 19.4 | 26.7 | 20.0 | 27 | M | 42 | 5484 | 3769 | 5053 | 8 | 376 | 368.33 | 0.0948 |
| Sep-16 | 23 | 19.4 | 26.7 | 20.0 | 1611 | F | 34 | 4250 | 2735 | 3667 | 14 | 269 | 394.56 | 0.0934 |
| Sep-16 | 23 | 19.4 | 26.7 | 20.0 | 1771 | F | 38 | 3981 | 2686 | 3601 | 10 | 304 | 396.01 | 0.1172 |
| Feb-17 | 9 | 3.3 | 15.6 | 0.0 | 1770 | M | 34 | 6143 | 4363 | 5848 | 5 | 328 | 337.53 | 0.0716 |
| Feb-17 | 9 | 3.3 | 15.6 | 0.0 | 1772 | F | 15 | 2799 | 1834 | 2459 | 12 | 172 | 360.96 | 0.0891 |
| Feb-17 | 9 | 3.3 | 15.6 | 0.0 | 27 | M | 43 | 5705 | 3950 | 5295 | 7 | 282 | 341.74 | 0.0680 |
| Feb-17 | 9 | 3.3 | 15.6 | 0.0 | 1611 | F | 35 | 4528 | 2968 | 3978 | 12 | 186 | 376.20 | 0.0595 |
| Feb-17 | 9 | 3.3 | 15.6 | 0.0 | 1771 | F | 39 | 4077 | 2715 | 3639 | 11 | 210 | 365.66 | 0.0736 |
| Oct-17 | 14 | 7.1 | 20.2 | 8.9 | 1770 | M | 34 | 5723 | 3873 | 5191 | 9 | 383 | 371.63 | 0.0942 |
| Oct-17 | 14 | 7.1 | 20.2 | 8.9 | 1772 | F | 15 | 2682 | 1699 | 2278 | 15 | 204 | 368.62 | 0.1144 |
| Oct-17 | 14 | 7.1 | 20.2 | 8.9 | 27 | M | 43 | 5480 | 3562 | 4775 | 13 | 343 | 371.39 | 0.0916 |
| Oct-17 | 14 | 7.1 | 20.2 | 8.9 | 1611 | F | 35 | 4424 | 2748 | 3683 | 17 | 214 | 377.51 | 0.0740 |

^a^ Estimates of TBW and FFM include gut contents
